# Statistical parametric and non-parametric control charts for monitoring residential water consumption

**DOI:** 10.1038/s41598-023-40584-w

**Published:** 2023-08-19

**Authors:** Allyson Belli Bogo, Elisa Henning, Andreza Kalbusch

**Affiliations:** College of Technological Science, Santa Catarina State University, Joinville, Brazil

**Keywords:** Environmental sciences, Engineering

## Abstract

The adoption of strategies for monitoring water consumption is essential for water resources management, contributing to the promotion of the sustainability in the water sector. Statistical process control (SPC) charts, which are widely used in the industrial sector, are statistical methods developed to improve the quality of products and processes. The application of this method has reached other areas over the last decades and has recently been identified as an option for environmental monitoring. In this context, the application of SPC charts emerges as an option for water consumption monitoring, whether in a building or an urban scale. Thus, this article aims to analyze the application of statistical process control charts in the monitoring of water consumption of two housing compounds in Joinville, southern Brazil. The methodological procedures include the use of the Shewhart and the EWMA control charts in addition to the non-parametric alternative, the EWMA-SN, assessing the effectiveness of these techniques in detecting water leaks in residential apartment buildings. The data sets, obtained through a telemetry metering system from the water utility, represent a period of 243 days. The results show that control charts are a powerful tool in identifying changes in water consumption patterns, with the EWMA chart flagging the leaks sooner.

## Introduction

The urbanization and industrialization processes are making the rise in water consumption and its consequent scarcity more evident^1^. Achieving sustainable water resource development is a globally important matter, especially in developing countries, where water-consumption-related issues are a growing challenge in terms of sustainability^2^. Monitoring water consumption allows the right decisions to be made regarding the management of water resources^3^ as it is useful to control water usage, detect leaks and control water waste^4^.

In the built environment, water and electricity data are used to make consumer behavior more efficient, improve demand forecasting, promote saving opportunities, investigate different customer segments, and potentially assist in the financial management of companies in the sector^5^. Smart water metering allows for the measurement, monitoring and analysis of water consumption at sub-daily intervals, with hourly, minute or second timescales, both at the household and at the water utility level^6,7^.

Most research works analyzing water consumption patterns focus on the domestic water consumption or water end-use^4,8,9^. Domestic water consumption refers to the consumption of housing units, while water end uses refer to the volume of water consumed by each type of individual plumbing fixture in the house^10^. It is also important to highlight that data access in urban centers is generally restricted and limited by consumer privacy protection measures, whether through data anonymization, access restriction or access control filters^7^. Few studies focus on telemetry processing and analysis of consumption data to improve the operation and maintenance of water supply systems^11^. The early identification of water losses in water supply systems, as well as the understanding of consumption patterns, is essential to promote adequate water usage management.

The primary causes of water loss in water supply systems are associated with leaks and bursts in the network and can be due to many factors, such as corrosion, stress induced by water-pressure variation, excessive surface loads, structural failures caused by ground movements or soil erosion, among many others^12^. Among the water-loss-related problems are the financial costs associated with unpaid water processing, repair costs, costs associated with service interruption, and intangible costs such as customer dissatisfaction and reduced water quality and public safety^12^. Therefore, leak detection and tracking become imperative actions for the competent authorities in the supplying of water services, and an important theme for research in water conservation field.

In the context of the water sector literature, several studies emphasize the importance of implementing reasonable and systematic strategies to manage water distribution networks. In a survey carried out with approximately 180 water utilities distributed in 14 countries, the need for leak-detection services was mentioned by most companies, however, only 40% reported having leak-control methods^13^. According to the authors, most utilities react to leaks by responding to obvious bursts and repairing the infrastructure as needed, with smaller or unreported leaks taking longer to correct, showing the need for more reasonable and systematic strategies for good infrastructure management. Many inspection methods, including electromagnetic, acoustic, ultrasonic, radiographic and thermographic have been proposed to detect leaks, but they do not enable long-term monitoring and require a specialized team to operate the equipment and interpret the readings^14^.

Leak-detection approaches can be broadly classified as model-based or real-process-data-based^15^. Model-based techniques must be validated in the field, with tests under typical operating conditions^16^. Data-based approaches are attractive alternatives which can yield accurate results^17,18^. Hu et al.^19^ performed a literature review on model-based and data-driven approaches for leak detection in water supply systems. They mention that model-based approaches require highly calibrated hydraulic models while data-driven approaches do not need an in-depth understanding of the water supply system. Hu et al.^19^ also state that model-based and data-driven approaches have advantages and limitations. A data-driven approach is appropriate when a large amount of historical data can be obtained^19^. Otherwise, when not much data is available and a hydraulic model can be obtained, model-based methods are preferred^19^. Wan et al.^20^ highlight that, for leak identification, data-driven methods can extract valuable information when there is a large amount of monitoring data and, for localization, model-based approaches can provide accurate leak location by using pressure data from multiple sensors. Driven by recent technological advancements in telemetry systems and water measuring equipment, research involving the monitoring of water supply systems has been exponentially emerging in the literature^21–23^. Those studies aim to help water utilities identify leaks and, consequently, reduce waste rates, representing an advance in terms of monitoring water losses in water supply systems.

In this context, statistical techniques are drawing attention because of the advantages related to their continuous application and simplicity in reading and interpreting data^24–26^. Statistical methods are tools in the diagnosis and optimization of the management and operation of various systems, from humans to the most complex physical systems^27^. Among these methods are statistical process control (SPC) charts. Control charts have the advantage of identifying situations in which a process may be behaving irregularly, allowing an investigation to be initiated on the cause and corrective actions to be taken^28^. Control charts are graphical analysis tools to identify outliers, such as leaks, in monitored data^19^.

In terms of application, control charts may be parametric or non-parametric. The main feature of the parametric charts relates to the statistical behavior of the process being monitored. Only when the data meet the assumptions of normality and independence can conclusions regarding the statistical state of the process be drawn^27^. Non-parametric control charts emerged as an alternative to such premises: their application does not depend on any specific assumption about the data distribution^29^.

In the built environment, monitoring water consumption using SPC techniques performs consistently well, potentially stimulating water conservation and the preventive management of water supply networks^30–32^. However, the application of control charts in this area is still uncommon and, from this perspective, progress is possible. Romano et al. ^31^ presented an SPC-based methodology to determine the approximate location of leaks. The methodology proposed provided water utilities with a wide range of benefits, making it possible to determine the area where a leak occurred, substantially reducing the necessary efforts and operational costs to track it^31^.

Water consumption may be associated with meteorological and climate variables over time^33–35^. Thus, water consumption data is not often normally distributed, which would be required for the application of parametric control charts. Therefore, non-parametric control charts are an alternative^29^.

In this context, this paper seeks to answer the following research questions:

1. Can statistical process control charts be used to monitor daily water consumption in buildings and, especially, to detect leaks?
2. Do parametric and non-parametric control charts perform differently when monitoring daily water consumption in buildings?

To answer the research questions, this work aims to analyze the application of statistical process control charts in the monitoring of water consumption of two housing compounds in the city of Joinville, southern Brazil. Additionally, parametric and non-parametric control charts are employed in a comparative case study to investigate their performance when applied to the monitoring of daily residential water consumption. This research contributes for the dissemination of the use of control charts to monitor and manage water resources, as there are few studies involving the application of parametric and non-parametric control charts for this purpose. Batista et al.^36^ mention the use of SPC at three levels of water management: macro, urban and building (or micro) level. The authors also suggest that studies at the micro level are scarce. In this sense, this study also presents a contribution to the development and application of SCC at the building level.

## Materials and methods

This paper applied Shewhart and the Exponentially Weighted Moving Average (EWMA) parametric control charts for individual measurements in addition to the non-parametric alternative, EWMA-SN, proposed by Graham et al.^37^, which involves signal statistics applied to an EWMA control chart. As a case study, the daily water consumption of two housing compounds in Joinville, Southern Brazil, was investigated to analyze the performance of control charts for the detection of leaks. In this section, we provide an overview of the statistical control charts (SCC) used in this paper and the main characteristics of the housing compounds from the case study.

### Statistical control charts (SCC): an overview

A control chart is basically composed of a central line (CL) that represents the mean value of the quality characteristic corresponding to the situation of the process under control and a pair of control limits: one of them located below the central line, called the lower control limit (LCL), and the other located above the central line, called the upper control limit (UCL) (Eqs. (1), (2) and (3) present the UCL, CL and LCL, respectively)^27^.

1 $$UCL={\mu }_{w}+L{\sigma }_{w}$$

2 $$CL={\mu }_{w}$$

3 $$LCL={\mu }_{w}-L{\sigma }_{w}$$

where ${\mu }_{w}$ corresponds to the mean; *σ*_*w*_ is the standard error and *L* is the distance factor from the control limits to the central line, expressed in standard error units. The measurements are plotted along the graph and, if any point is beyond the control limits, the process is said to be out of statistical control and it is suggested that the causes be investigated^27^.

The statistical control chart performance can be expressed by the average run length (ARL), which is the average number of samples required to signal that an event is out of statistical control^38^. As stated by Kostyszyn et al.^38^, an effective control chart has a large ARL under statistical control conditions and a small ARL when the process is out of control. According to Sancho et al.^39^, this theory is associated to a Type I error (false alarm rate) and a Type II error (false negative) and, when the statistical power of the SCC is increased to signal out-of-control events, so is the false alarm rate.

The development of a control chart is divided in two phases. Phase 1, which corresponds to the chart parameters’ estimation, should only be ended when the process is stable^27^. The assumptions of data normality and independence must be verified for the application of the parametric charts. In Phase 2, the limits calculated in Phase 1 are used to monitor the process^27^.

The control chart parameters are estimated for the desired in-control ARL^40^. In this research, the statistical parameters of the control charts were set so that they all presented ARL_0_ ≅ 370, that is, a false alarm rate (type I error) α = 1/370. This definition is widely used in industrial settings and helps to compare between control charts^40,41^.

The Shewhart control chart is the most popular SCC^40^. From Eqs. (1) and (3), to estimate the control limits of Shewhart control chart, the moving range of two consecutive readings (*x*) is used as a base for process viability assessment. The moving range (MR) is calculated as ${MR}_{i}=\left|{x}_{i}-{x}_{i-1}\right|$, where *i* is an integer representing the observation point. Thus, the Shewhart control chart for individual measurements to monitor the mean has the upper limit, central line and lower limit calculated as shown in Eqs. (4), (5) and (6)^27^:

4 $$UCL=\overline{x}+3\overline{MR}/{d}_{2}$$

5 $$CL=\overline{x}$$

6 $$LCL=\overline{x}-3\overline{MR}/{d}_{2}$$

where $\overline{x}$ is the average of all the individual observations; $\overline{MR}$ is the average of all the moving ranges and ${d}_{2}$ is a defined constant that depends on the number *n* of observations. For *n* = *2*, ${d}_{2}$ is 1.128.

In the Shewhart chart control technique, the decision regarding the process state of control at any time depends solely on the most recent measurement from the process. According to Montgomery^27^, Shewhart control charts are effective in detecting large changes. To improve the sensitivity of the control chart to small process shifts, some techniques have been proposed, such as the Western Electric Run Rules and the Exponentially Weighted Moving Average (EWMA)^42^.

The Western Electric Run Rules, also called supplementary run rules^27^, aim to detect small changes in the average, since they could yield points further from the center still within the control limits, which would go undetected^43^. The process is classified as out of statistical control if (1) one point is out of the 3*σ* control limit, (2) two out of three consecutive points are beyond the 2*σ* limits, (3) four out of five consecutive points are 1*σ* or further away from the central line and (4) eight consecutive points are on the same side of the central line. These rules let smaller process changes be detected more quickly. However, using them yields more false alarms^44^.

As an alternative, EWMA (Exponentially Weighted Moving-Average) control charts are an effective choice to detect small shifts in the process^45^. For the EWMA control charts, the decision depends on the EWMA statistic, which is an exponentially weighted average of all prior data, including the most recent measurement. By the choice of a weighting factor (λ), the EWMA control chart can be made sensitive to a small or gradual drift in the process, whereas the traditional Shewhart control chart can only react when the last data point is outside a control limit^46^.

The EWMA statistics can be defined through Eq. (7) ^27^, in which λ represents the weighting factor attributed to the observations.

7 $${z}_{i}=\lambda {x}_{i}+\left(1-\lambda \right){z}_{i-1}$$

where *z*_*i*_ is the current moving average weight, *x*_*i*_ is the current sample value, *i* is an integer representing the current index and 0 < λ < 1 is the smoothing constant. The value of *z*_*0*_ is the process target, which can be the process average. If observations *x*_*i*_ are independent random variables with variance σ^2^, then the variance of *z*_*i*_ is given by Eq. (8) ^27^.

8 $${\sigma }_{{z}_{i}}^{2}={\sigma }^{2}\left(\frac{\lambda }{2-\lambda }\right)\left[1-{\left(1-\lambda \right)}^{2i}\right]$$

Thus, the EWMA chart control limits are found based on the asymptotic *z*_*i*_ variance. So, once the process standard deviation is estimated, the control limits, derived from Eqs. (1), (2) and (3), are represented by Eqs. (9), (10) and (11) ^27^.

9 $$UCL={\mu }_{0}+L\sigma \sqrt{\frac{\lambda }{\left(2-\lambda \right)}\left[1-{\left(1-\lambda \right)}^{2i}\right]}$$

10 $$CL={\mu }_{0}$$

11 $$LCL={\mu }_{0}-L\sigma \sqrt{\frac{\lambda }{\left(2-\lambda \right)}\left[1-{\left(1-\lambda \right)}^{2i}\right]}$$

with *μ*_*0*_ approximated by the sample average $\overline{x}$; L is the extension of the control limits, that is, the multiple of standard deviation that determine the distance from the control limits to the central line.

For the non-parametric EWMA-SN chart, where *x*_*i*_ is the individual measurement of an *i*-sized process with a cumulative distribution function *F* and median *θ* being monitored, the signal statistics are defined through Eq. (12) ^37^.

12 $${SN}_{i}=sign\left({x}_{i}-{\theta }_{0}\right)$$

where *i* is an integer representing the observation index and *θ* is the specified median to be monitored. The variable *sign* is attributed to the current observation in relation to a pre-defined parameter, found through Eq. (13), assuming a value of 1, 0 or − 1 for each observation^37^.

13 $$ sign\left( x \right) = \left\{ {\begin{array}{*{20}l}    1 \hfill & {if\;x > 0} \hfill  \\    0 \hfill & {if\;x = 0} \hfill  \\    { - 1} \hfill & {if\;x < 0} \hfill  \\   \end{array} } \right. $$

The non-parametric EWMA-SN chart plotting statistics is obtained through a sequential accumulation of the *SN*_*i*_ signal statistics, and is defined by Eq. (14) ^37^.

14 $${Z}_{i}=\lambda S{N}_{i}+\left(1-\lambda \right){Z}_{i-1}$$

where 0 < λ < 1 is the smoothing constant and *Z*_*0*_ = 0. The *Z*_*i*_ plotting statistics standard deviation is found through Eq. (15)^37^.

15 $${\sigma }_{{Z}_{i}}=\sqrt{\frac{\lambda }{2-\lambda }\left(1-{\left(1-\lambda \right)}^{2i}\right)}$$

Hence, the central line and the upper and lower control limits of the non-parametric EWMA-SN control chart for the median are given by Eqs. (16), (17) and (18)^37^.

16 $$UCL=L\sqrt{\frac{\lambda }{2-\lambda }\left(1-{\left(1-\lambda \right)}^{2i}\right)}$$

17 $$CL =0$$

18 $$LCL=-L\sqrt{\frac{\lambda }{2-\lambda }\left(1-{\left(1-\lambda \right)}^{2i}\right)}$$

where *L* is the distance from the control limits to the central line.

The main characteristics, strengths and weaknesses of the three applied graphs are summarized in Table 1.

**Table 1:** Summary of the parameters, characteristics, strengths and weaknesses of Shewhart, EWMA and EWMA-SN control charts.

Control Chart	UCL	CL	LCL	Assumptions	Strengths	Weaknesses
Shewhart^a^	Equation 4	Equation 5	Equation 6	Data must be independent (absence of autocorrelation) and follow a Normal Distribution	Efficient to detect large changes in the mean of a process	Less sensitive in signaling small magnitude changes (< 1.5 standard errors)
						Does not consider the history of the process
EWMA^a^	Equation 9	Equation 10	Equation 11	Data must be independent (absence of autocorrelation)	Efficient to detect small and persistent changes in the mean of a process	Less sensitive in detecting large changes
				Shows some robustness to non-normality	Provides a forecast of the process mean over the next period	
EWMA-SN^b^	Equation 16	Equation 17	Equation 18	Independence (absence of autocorrelation)	Efficient to detect small and persistent changes in the median of a process	Less sensitive in detecting large changes
					Useful when the information about the form of the underlying distribution is limited	

^a^ Montgomery^27^.

^b^ Graham et al.^37^.

### Case study

**Figure 1:**
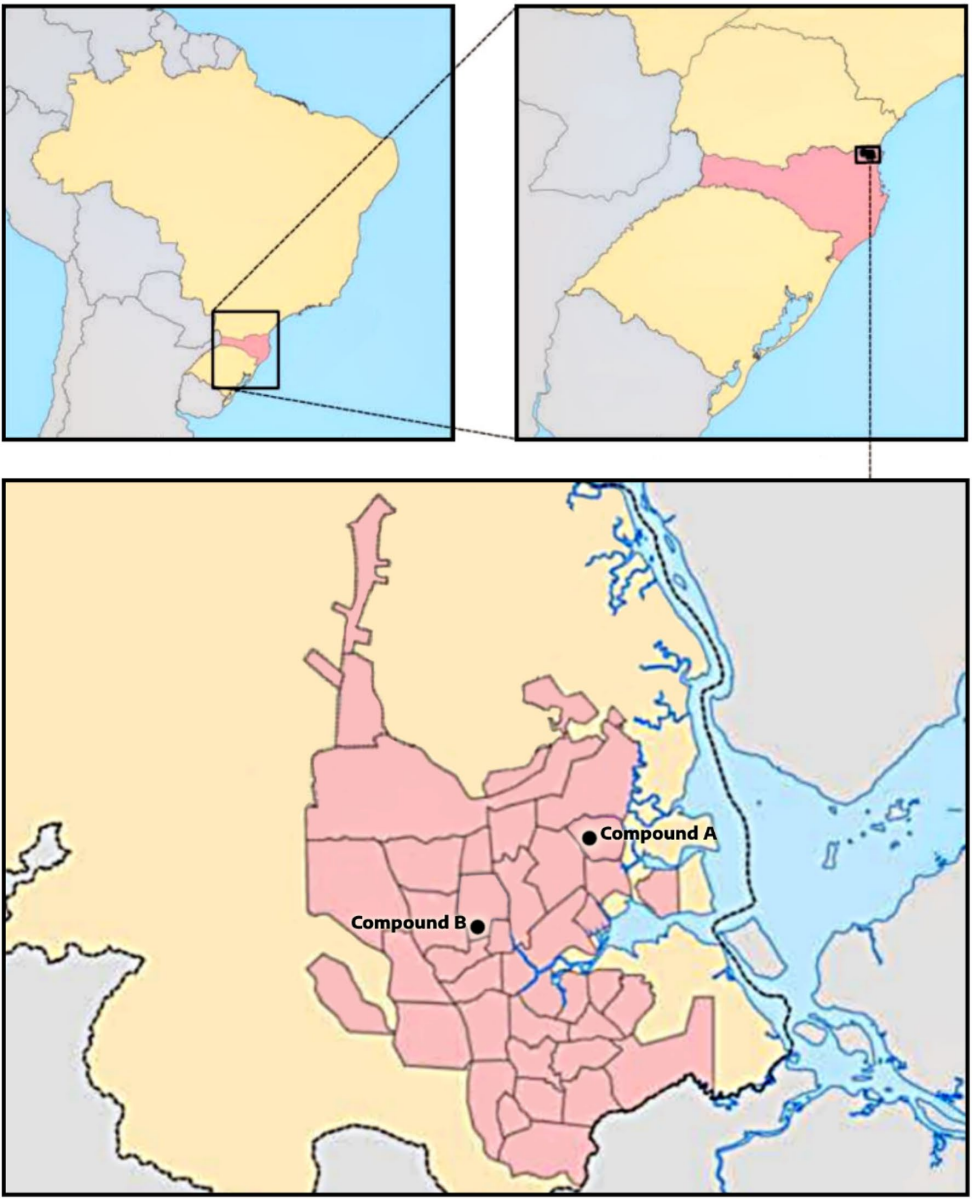
Geographic location of the housing compounds in Joinville, southern Brazil. Adapted from^50^.

As a case study, the daily water consumption of two housing compounds in Joinville, Southern Brazil, was investigated to analyze the performance of control charts in the detection of leaks. Figure 1 shows the location of the two housing compounds in the city of Joinville. Compound A is located in a neighborhood where the average monthly income per capita is 1.52 minimum wages and the demographic density is 7622 inhabitants/km^2^^47^. Compound B is located in a neighborhood where the average monthly income per capita is 5.74 minimum wages and the population density is 2742 inhabitants/km^2^^47^. Based on the number of residents per neighborhood in the last census from Instituto Brasileiro de Geografia e Estatística [Brazilian Institute for Geography and Statistics]^48^, the estimated number of residents in compounds A and B are 1088 and 29, respectively.

The water consumption data were obtained from Joinville's water utility company. These data are acquired through a telemetry system, which captures water consumption values at sub-daily intervals from each consumer unit and sends them to a database via radio. Consumption data were obtained at hourly intervals using water meters with a telemetry system equipped with radio transmitters installed in the water supply system of the compounds. Although the data are available on an hourly basis, the analyses were carried out considering the daily consumption. As a retrospective study, data previously measured by the system were analyzed. This is a retrospective analysis^49^, as water consumption data were collected after leaks occurred. In order to guarantee the privacy and security of the personal and private information of each unit, the two residential complexes were called housing compound A and housing compound B. Both were chosen as they had water leakages previously detected by the telemetry system in the past. The housing compound A is composed of low-income units, with 16,989.01 m^2^, distributed in 20 4-story buildings with 16 apartments per floor, totaling 320 apartments. According to data from the water utility, the housing compound A had water leakage between the months of September and November 2021. Housing compound B has a total area of 15,566 m^2^, distributed in 11 high-standard residences. The water leak in this complex occurred between May and August 2021.

Missing values were linearly interpolated. Then, based on the retrospective data series, the definition of periods for Phases 1 and 2 of the control charts' construction was investigated. For Phase 1, which corresponds to diagnosis, assumption checking and parameter estimation, a data set was defined under stable and representative conditions of the process under control. The check for normality was performed through the Shapiro–Wilk hypothesis test and the independence through the autocorrelation test with a significance level of $\alpha =5{\%}$. The rest of the series was used for Phase 2, called monitoring. At the end of this stage, the defined time series represented the daily water consumption data between 04/01/2021 and 11/29/2021, totaling 243 days, with Phase 1 being delimited by the first 30 days of the series (April 2021).

Then, the results obtained from the application of parametric and non-parametric control charts in the different series were compared, assessing the change detection speed in the water consumption pattern, false alarms, and the effect of seasonality in their application. When the goal is to compare the control charts’ performances, a commonly used parameter is the Average Run Length (ARL). All charts will be constructed for the same value of ARL_0_ (370) and analyzed with respect to the speed of detection of a true alarm.

**Figure 2:**
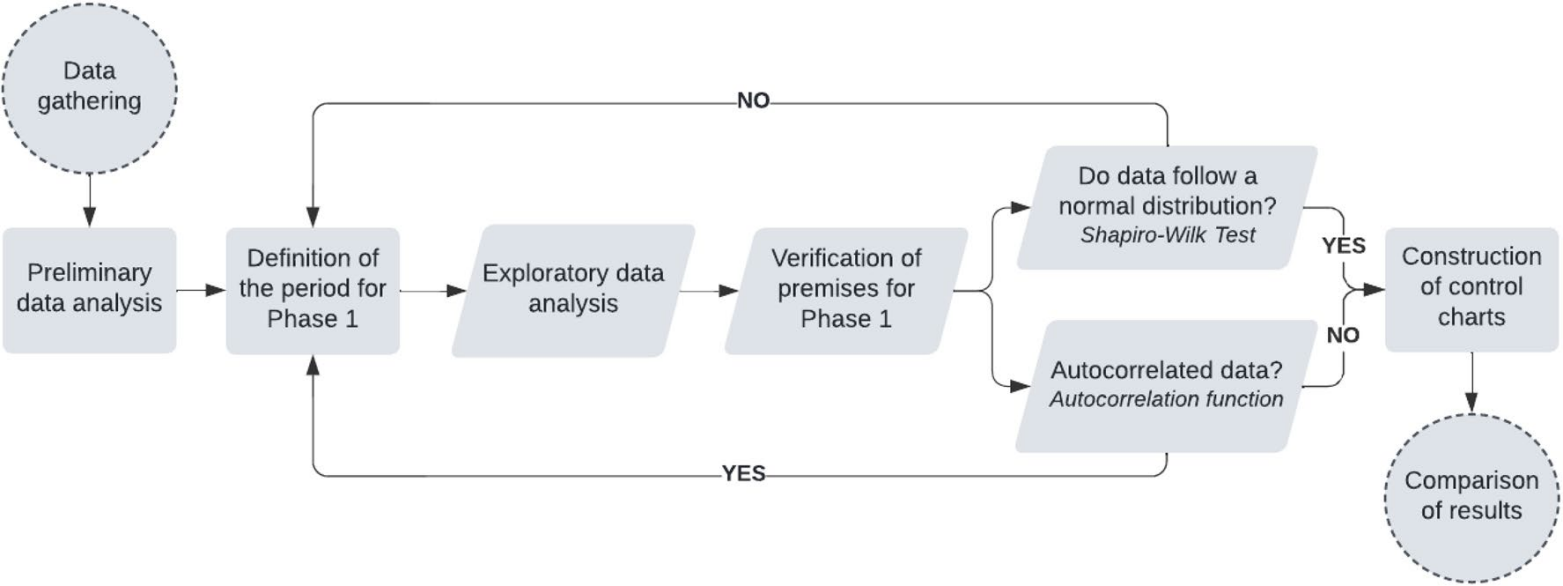
Methodological procedures.

The methodological procedures used to conduct this research were divided as shown in Fig. 2.

All analyses and applications were performed using the R software^51^ with the packages qcc^52^, ggplot2^53^, forecast^54^ and imputeTS^55^. A routine was developed in R language for the non-parametric EWMA-SN chart.

## Results

Based on the proposed methodology, the water consumption data from two housing compounds were analyzed. Table 2 presents the descriptive statistics of the consumption data of the two housing compounds. The results obtained from the descriptive analyses suggests the existence of water consumption pattern changes through the time series. These events might indicate water leaks, but they can also be related to meteorological and climate variables, such as weather changes and precipitation, which can influence water demand^33–35^.

**Table 2:** Descriptive statistics of water consumption (in m^3^/day) for the two residential condominiums.

Description	Mean (m^3^/day)	Standard deviation (m^3^/day)	Variation coefficient (%)	Minimum (m^3^/day)	Median (m^3^/day)	Maximum (m^3^/day)
Housing compound A	153.60	36.13	23.52	83.30	141.10	330.70
Housing compound B	20.19	7.87	38.96	4.10	20.90	44.00

**Figure 3:**
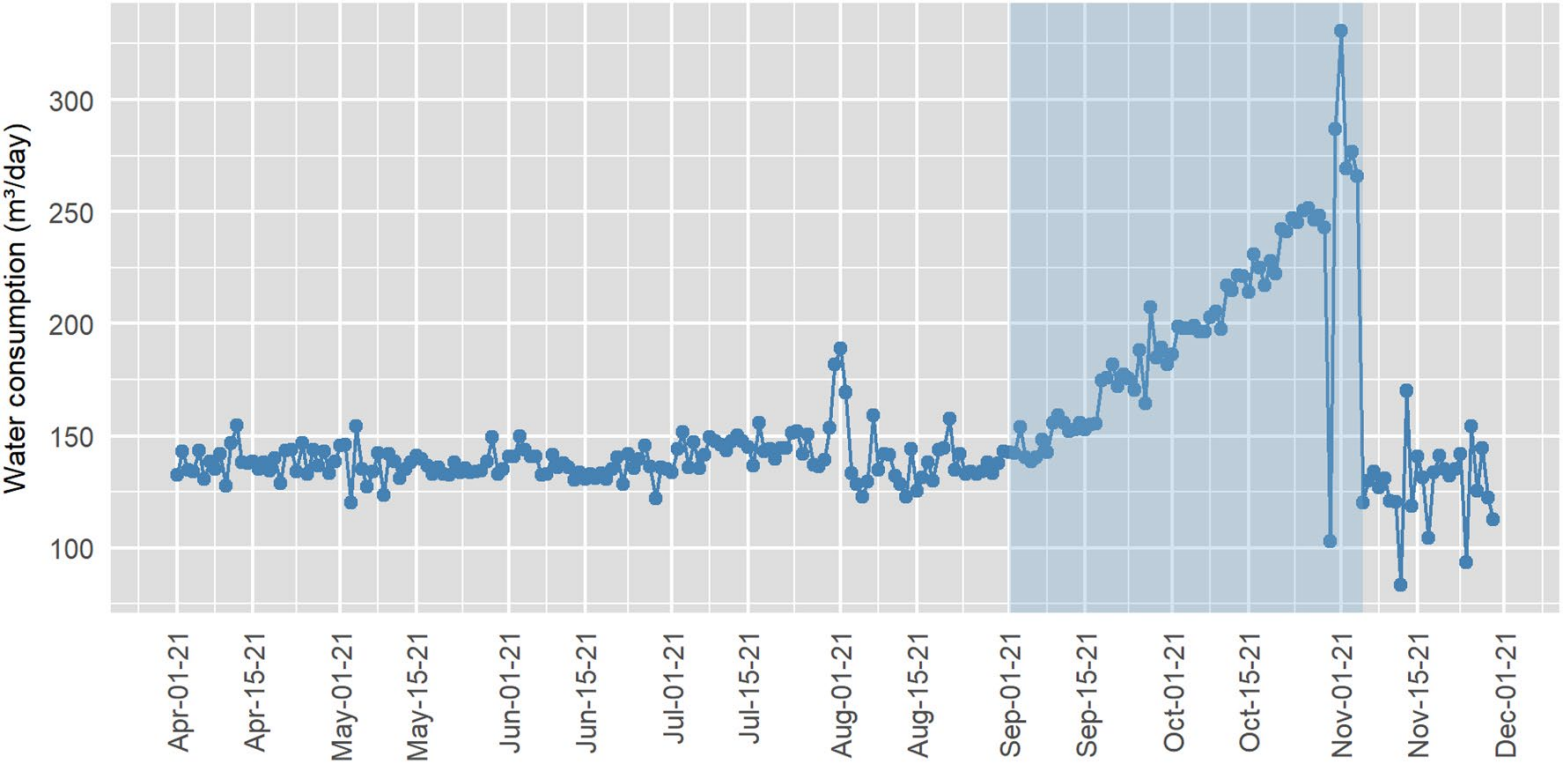
Time series of water consumption in the housing compound A.

The average daily water consumption per residential unit is 480 L/household/day in compound A and 1835 L/household/day in compound B. Based on the estimated population, the average per capita consumption in compounds A and B are 141.2 and 695.8 L/person/day, respectively. These numbers highlight the differences in water consumption in the objects of study, since compound A is composed of low-income units and compound B is a condominium of 11 high-standard residences. The time series chart for the housing compound A (Fig. 3) shows the occurrence of a water leakage highlighted in the chart, between the months of September and November 2021. This period is characterized by the expressive increase in the value of the observations.

**Figure 4:**
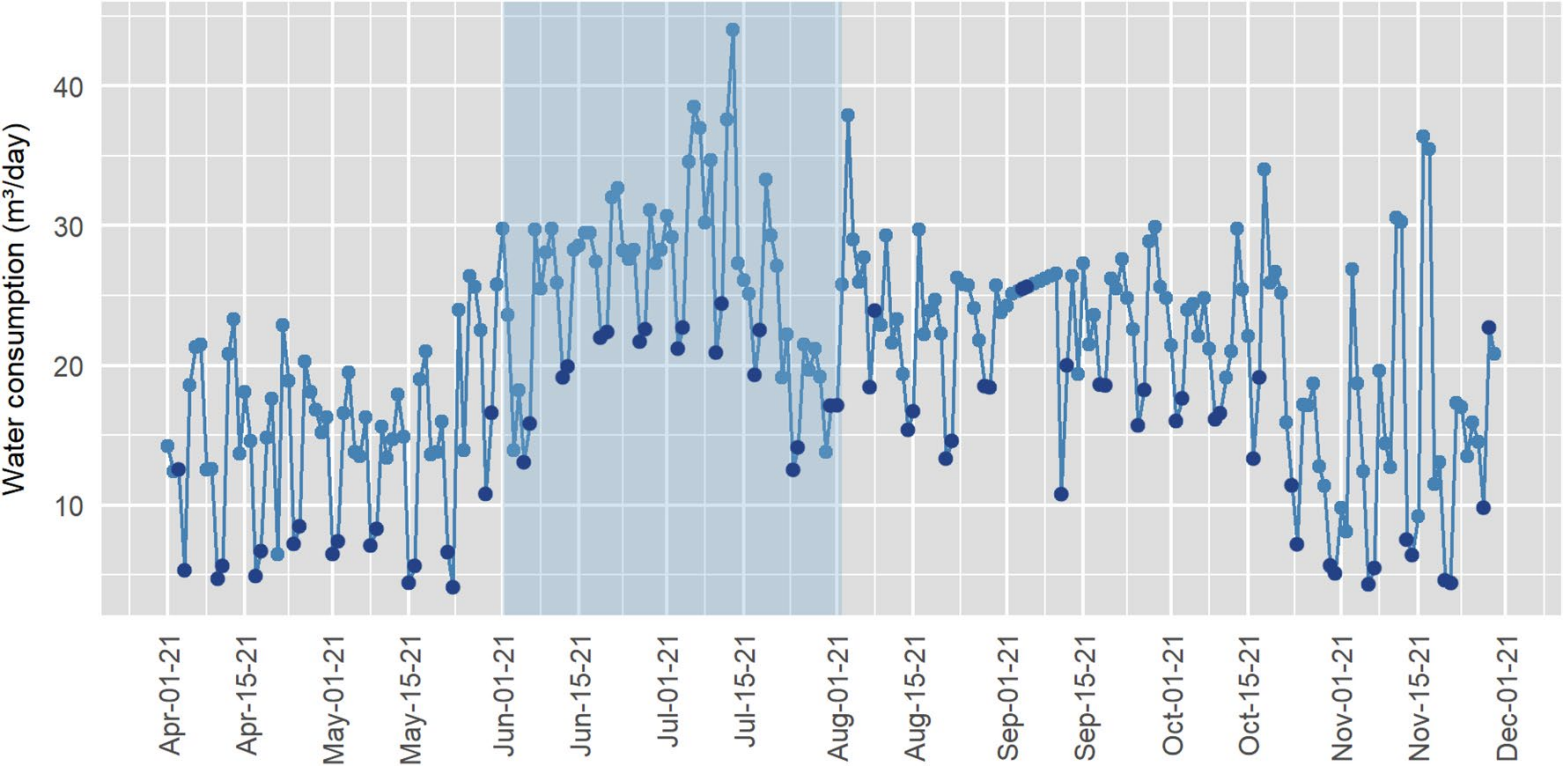
Time series of water consumption in the housing compound B.

The time series chart for daily water consumption of the housing compound B is shown in Fig. 4. The chart highlights the occurrence of a water leakage between the months of June and August 2021. The water consumption pattern in compound B presents stability in the weekly periods, staying consistent during business days, with a drop on the weekends, which are indicated with the dark blue dots in Fig. 4.

Regarding the fulfillment of the necessary assumptions for the application of the parametric charts, no series presented autocorrelation problems and the normality assumption was met, for the two housing compounds data (*p*-value = 0.53 and *p*-value = 0.071).

### Control chart application

**Figure 5:**
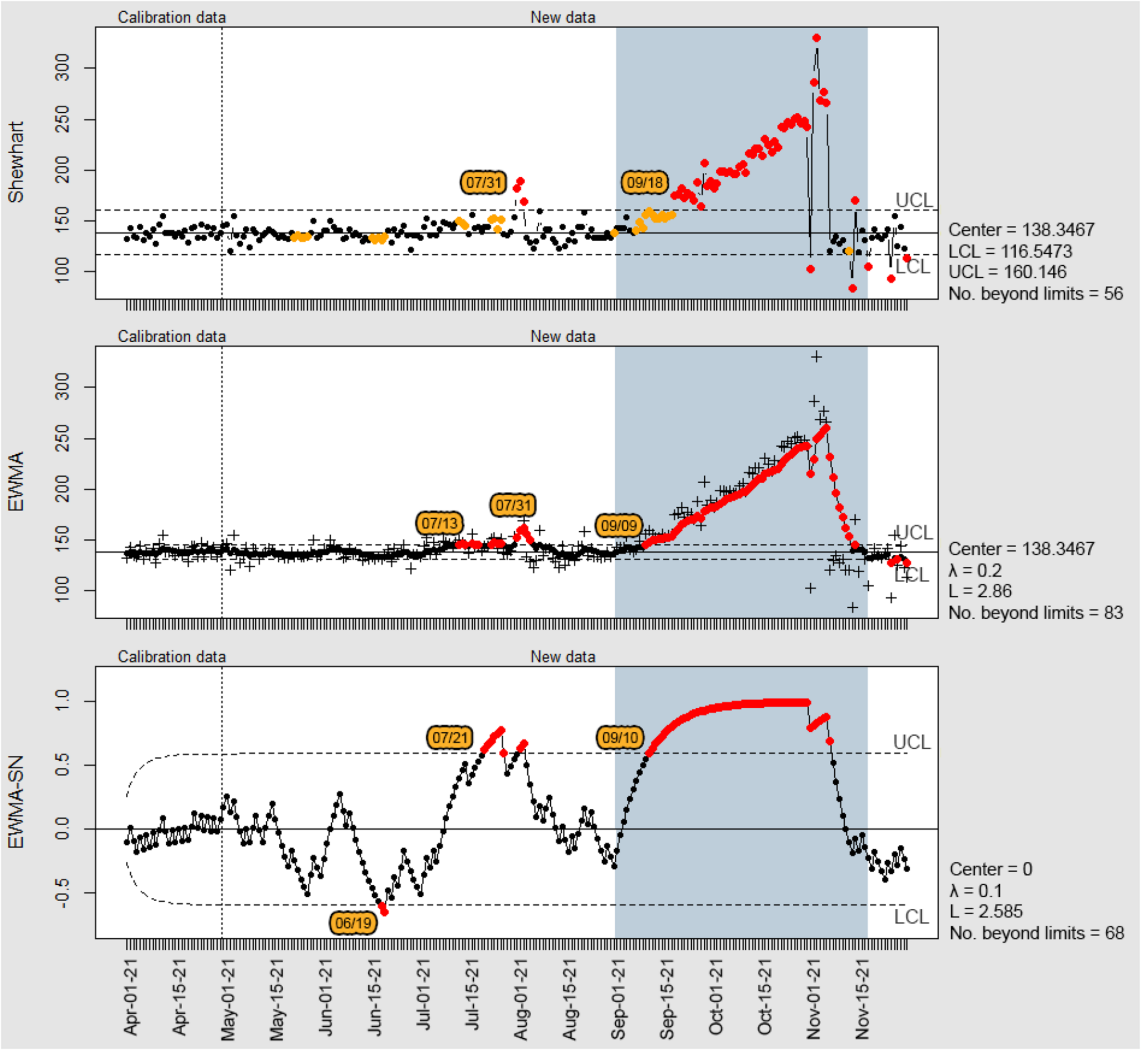
Control Charts for the housing compound A water consumption data.

Figure 5 shows the results obtained from the application of Shewhart, EWMA and EWMA-SN control charts to monitor the housing compound A. The period in which the leak occurred is indicated in light blue. The Shewhart control chart flagged 56 out-of-statistical-control points, with 3 observations at the beginning of August and the others starting from 09/18/21, which corresponds to the period of the water leak. The chart indicates the leak on 09/18/21, but the supplementary rules show signs of an increase in the average starting from 09/06/21. The first observations plotted beyond the upper control limit may be related to changes caused by external variables. Regarding the second half of October, after the water distribution network was repaired, the observations plotted beyond the lower and upper control limits may represent a maintenance in the metering system, which could be verified by the water utility. During this period, the data acquisition was interrupted, and the subsequent measurement accumulated the consumption of the previous day, indicating significant changes in the process.

The EWMA chart flagged a leak on 09/09/21, detecting it faster than the Shewhart chart, without the application of supplementary rules. Using the rules implies changing the ARL_0_, which could yield false alarms. The EWMA chart also showed two periods with points above the UCL starting on 07/13/21, in which the process accumulated consecutive observations above the CL. After the occurrence of the water leak, the magnitude of the observations also resulted in false alarms due to the chart assigning a weight to the recent readings.

The non-parametric EWMA-SN chart flagged the water leak on 09/10/21 and behaved similarly to the EWMA due to their similar plot statistic calculations. The difference is the indication of two observations below the LCL in the month of June, easily suppressed with the adjustment of the control limits, as well as a greater chart stability after the occurrence of the water leak, not affected by the likely maintenance of the metering system.

**Figure 6:**
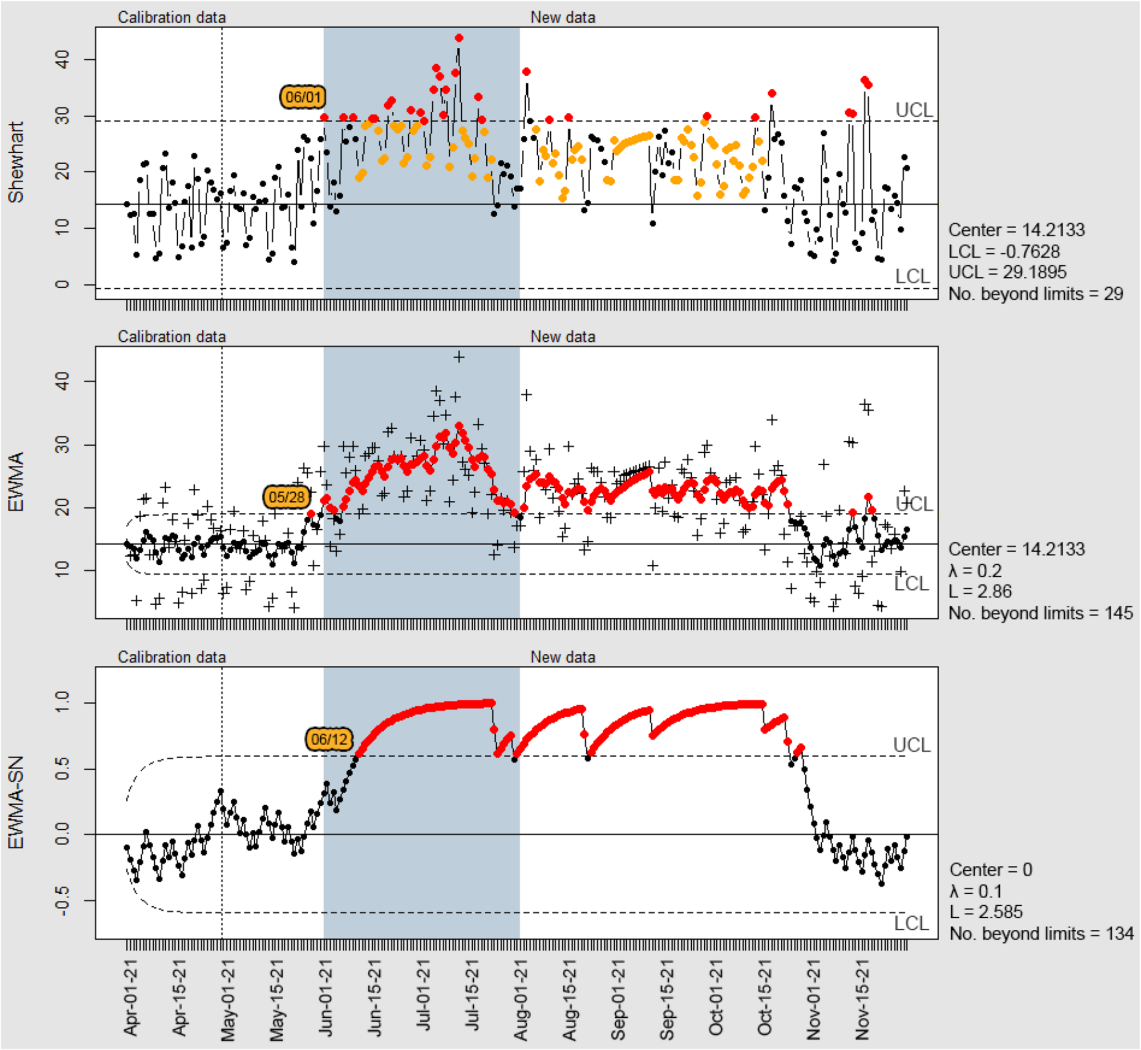
Control Charts for the housing compound B water consumption data.

Figure 6 shows the Shewhart, EWMA, and EWMA-SN control charts to monitor water consumption in housing compound B, with the period when the leak occurred indicated in light blue. The construction of the Shewhart control chart for compound B resulted in 29 observations plotted beyond the control limits, indicating that water consumption was out of statistical control at these points. In addition to the conventional analysis of points exceeding control limits, the chart also flagged periods when the process violated Western Electric's set of rules, mainly concerning plotting points on the same side of the central line or at least 1σ apart.

The Shewhart chart flagged a change in the process as of 06/01/21, corresponding to the period of the water leakage informed by the water utility company. As of September, the points plotted beyond the UCL correspond to isolated dates and may be related to changes caused by other factors. These observations present lower magnitude and recurrence in relation to the period of occurrence of the water leakage.

Regarding the EWMA control chart, its application yielded 145 points marked beyond the UCL, concentrated mainly in the interval during the water leakage, but extending until the month of October, mainly motivated by the weight assigned to old observations in the plotting chart statistics. This characteristic also contributed to an increase in the false alarms, as can be observed from the month of September on. This period does not correspond to the occurrence of a water leak, however minor changes in water consumption were flagged. As Montgomery^27^ points out, this is a characteristic of the EWMA control chart, being widely used for the detection of small changes in the process. The EWMA chart flagged the water leakage in compound B more quickly than the Shewhart chart, starting on day 05/28/21.

The non-parametric EWMA-SN chart showed analogous behavior to the EWMA in signaling false alarms, flagging 134 observations beyond the control limits. Although this technique presents the advantage of not requiring the actual numerical value of the observations, only the indication of whether they are greater or less than an established parameter of interest, this characteristic resulted in a delay in signaling a change in the process, since the magnitude of the observation being plotted is not considered. The EWMA-SN chart signaled the escape from statistical control on 12/06/21, representing a delay of approximately two weeks in relation to the other techniques.

In both compounds, the EWMA chart obtained the best results in water leak detection speed. Although the Shewhart chart detected the leak sooner considering the Western Electric rule set, the use of these rules implies in a change of ARL_0_, reflecting in a higher signaling of false alarms. The use of the median as the reference value for the EWMA-SN chart resulted in greater graph robustness to sudden changes in the process. This aspect decreased alarm signaling, delaying the identification of the water leakage in compound A. In compound B, this behavior was not observed, since the signaling of the water leakage occurred after a period of successive observations above the CL (Central Line).

In both compounds, the water leaks were preceded by a sequence of observations above the CL, rather than a sudden change in the process, suggesting that investigating small, consistent changes in the water consumption pattern can help identify leaks. These results agree with the work conducted by Wan et al.^56^, who stated that gradual leakages could be more challenging to detect due to the small amplitude and the slow increase trend at the start.

This study evaluated the monitoring of water consumption at a building scale, however the application of control charts at an urban scale is also possible and proved to be effective in the studies by Jung et al.^30^ and Wan et al.^56^. Wan et al.^56^ proposed an online system on leakage detection based on EWMA-enhanced Tukey method. The effectiveness of the proposed system was demonstrated in a simulated hypothetical town under various scenarios^56^. Jung et al.^30^ mention that a pipe burst leads to water loss, higher flows and lower pressures in the system. In this sense, early detection reduces the failure duration, increasing system resilience and maintaining system functionality^30^. These conditions apply to both the urban and building scale.

## Conclusions

This paper presents a contribution to the application of statistical control charts in a case study on water consumption in residential buildings, highlighting the ability of these charts when used to detect leaks. Shewhart and EWMA parametric process statistical control charts and the EWMA-SN non-parametric chart were applied to monitor water consumption in real water consumption data from two housing compounds. The performance of the graphs was compared by observing the speed in signaling existing leaks in both residential complexes.

The results show the traditional Shewhart and EWMA charts performed the best, simultaneously detecting the water leak, while the non-parametric EWMA-SN chart could not flag the leak. Using the median as the EWMA-SN chart reference value yielded greater robustness for large process changes, not detecting observations found in the other ones.

The analyses performed in this research were limited because the data studied represent a retrospective time series of the housing compounds’ total water consumption, where detailed information about each household's individual consumption was not available. Thus, it was not possible to identify if the changes in consumption happened in a specific household, which could represent the occurrence of a water leakage in the consumer unit in question, or if the changes signaled in the graphs represent changes in the water consumption pattern of the compound as a whole.

The methodology proposed in this research can help in the development of automated monitoring tools for the water consumption in a building or a city. Control charts are powerful tools for process monitoring that allow tracking and flagging erratic behaviors. The combination of these techniques can reduce false alarms, simplifying its application and analysis interpretation. The findings of this study may fill important knowledge gaps about the use of control charts to monitor household water consumption. The application of these techniques can speed up the detection of water leaks, generating alarms that signal the need to take immediate action to correct the cause of the leak. In addition, the results show that control charts can be used to monitor water consumption continuously and remotely via IoT, allowing for reduced water waste and intervention time to correct water leaks. The control charts can also be used to monitor the pattern of water demand over time, assisting in the development of public policies aimed at water conservation at times or periods of peak consumption.

The limitations of this work are also opportunities for future research. A suggestion is to compare the performance of control charts with other methods of monitoring water consumption in building systems, to detect leaks and other changes in consumption patterns. The influence of weather on the historical series and the impact of seasonality on the leak detection capacity of the control charts could be evaluated in future studies, since many cities in Brazil have digitalized historic weather records.

To further minimize false alarms, specific ARL_0_ values should be identified to monitor water consumption. It is advisable to estimate ranges of ARL_0_ values corresponding to different data acquisition frequencies, such as daily or hourly. In addition, considering the possible seasonality present in water consumption patterns, studying the adaptation of these charts to handle this underlying structural variation is suggested. Also, as stated before, leveraging the potential of the EWMA chart to forecast the process mean should be investigated, aiming to improve its predictive capacity. Finally, evaluating the use of combined Shewhart-EWMA charts emerges as an alternative worth considering, as it has the potential to enhance the performance of EWMA charts in detecting larger changes.

Using control charts to monitor water consumption allows to determine if changes in legislation or educational campaigns had an impact on reducing water waste. Control charts can also be used to investigate the effects of water saving, as well as to identify leaks in water supply systems. The use of control charts to monitor a building’s supply system can also be implemented from applications developed to analyze data collected from water meters equipped with a telemetry system. These methods can be programmed in any computational language and even spreadsheets can be used due to the simplicity of implementation. This context justifies the development of new methodologies to monitor water consumption using control charts.

## Data Availability

The data that support the findings of this study are available from Companhia Águas de Joinville, but restrictions apply to the availability of these data, which were used under license for the current study, and so are not publicly available. Data are however available from the authors (elisa.henning@udesc.br) upon reasonable request and with permission of Companhia Águas de Joinville.
